# Myositis ossificans circumscripta after surgery and radiotherapy and during sunitinib treatment: a case report

**DOI:** 10.1186/s13256-022-03664-5

**Published:** 2022-12-07

**Authors:** Pierre-Yves Cren, Nicolas Penel, Abel Cordoba, Gauthier Decanter, Louise Gaboriau, Mariem Ben Haj Amor

**Affiliations:** 1 Département d’oncologie Médicale, Centre Oscar Lambret, 3 Rue Frédéric Combemale, 59000 Lille, France; 2 Université de Lille, Lille, France; 3 Département de Radiothérapie, Centre Oscar Lambret, Lille, France; 4 Département de Chirurgie, Centre Oscar Lambret, Lille, France; 5 Centre Régional de Pharmacovigilance, Service de Pharmacologie Médicale, CHU Lille, Lille, France; 6 Département d’imagerie Médicale, Centre Oscar Lambret, Lille, France

**Keywords:** Myositis ossificans circumscripta, Alveolar soft part sarcoma, Sunitinib, Magnetic resonance imaging, Computed tomography, Plain radiograph, Pharmacovigilance

## Abstract

**Background:**

Myositis ossificans circumscripta is a self-limiting, benign, ossifying lesion that can affect any type of soft tissue. It is most commonly found in muscles as a solitary lesion. A history of recent trauma has been reported in approximately 50% of cases. Clinically, MOC presents as a painful swelling, which rapidly increases in size. The pain and inflammatory symptoms spontaneously disappear after approximately 2–6 weeks, and the mass stabilizes or decreases. Radiologically, myositis ossificans circumscripta can be divided into two phases. The first is the acute phase, which is followed by the mature phase 2–6 weeks later. During the acute phase, the radiological aspect does not show any specific abnormality. In the mature phase, plain radiographs and computed tomography show blurred calcifications around a hypodense center. We describe here the first case of myositis ossificans circumscripta, with appropriate follow-up, occurring during sunitinib exposure.

**Case presentation:**

We report a case of myositis ossificans circumscripta in a 34-year-old man (ethnicity unknown) receiving sunitinib for metastatic alveolar soft part sarcoma of the left thigh after surgery and radiotherapy. Four months after the first dose of sunitinib, the patient experienced painful swelling in the surgical scar area. Magnetic resonance imaging showed diffuse and marked edema of the anterior compartment of the thigh, without nodular lesions circumscribing a central core, and without bone signal abnormality. The increased visibility of the intermuscular fascia and convergence of normal muscle fibers (black hole effect), without the displacement seen in tumors, were suggestive of myositis. Therefore, antiangiogenic treatment was discontinued, and the symptoms rapidly resolved within a few days. Three weeks after the discontinuation of sunitinib, the inflammatory findings completely disappeared. Two months after the diagnosis of myositis ossificans circumscripta, plain radiographs and computed tomography showed an extensive calcified mass measuring > 12 cm. The continuation of favorable clinical outcomes was confirmed.

**Conclusions:**

To the best of our knowledge, this is the first case of myositis ossificans circumscripta with appropriate follow-up occurring during sunitinib exposure. Owing to multimodal treatment of sarcoma, we cannot rule out the radiotherapy and surgery causality.

## Background

Myositis ossificans circumscripta (MOC) is a self-limiting, benign ossifying lesion that can affect any type of soft tissue, including subcutaneous fat, tendons, and nerves. However, it is most commonly found in muscles as a solitary lesion [1, 2].

Herein, we report the case of a patient who developed MOC during treatment with sunitinib after surgery and radiotherapy for metastatic alveolar soft part sarcoma (ASPS).

## Case presentation

**Fig. 1:**
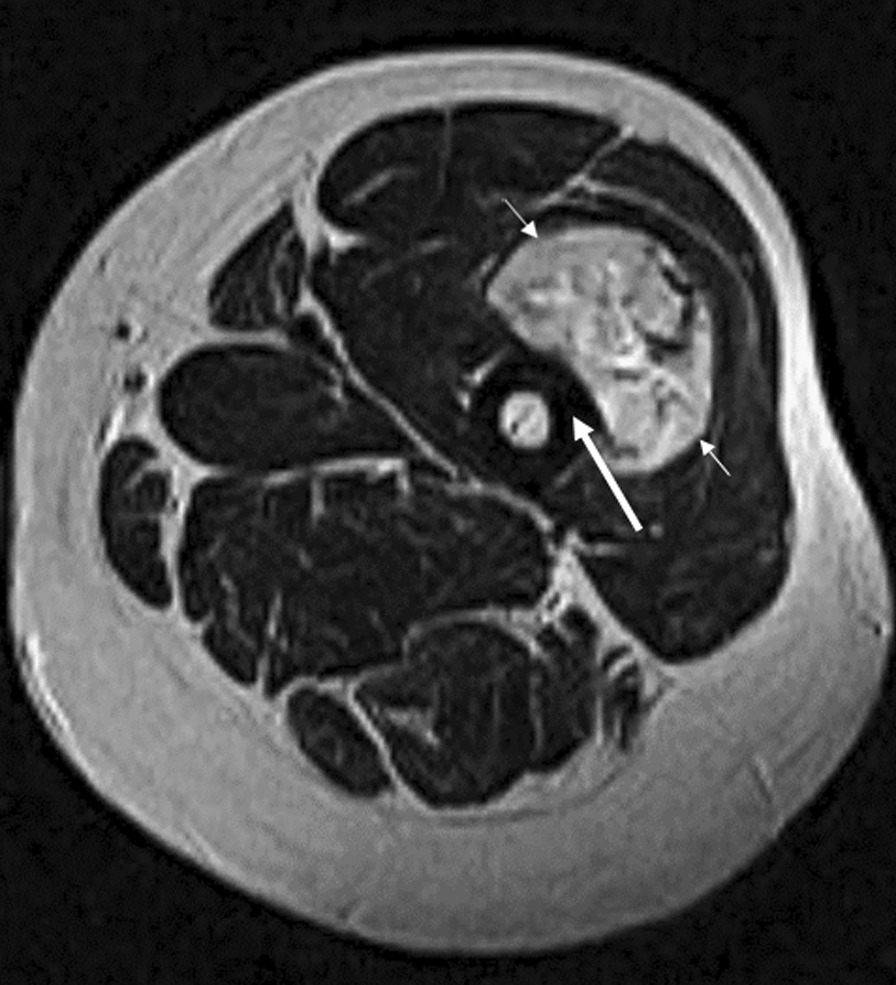
Axial T2-weighted magnetic resonance (MR) image showing a deep mass (short arrows) with high signal intensity corresponding to the primitive lesion (ASPS). The lesion is in contact with the cortical bone without edema or osteolysis (long arrow) *MR* magnetic resonance, *ASPS* alveolar soft part sarcoma

A 34-year-old man (ethnicity unknown) with a history of childhood eczema and asthma presented with an ASPS of the left thigh (10 cm) in June 2015 (Fig. 1). He underwent neoadjuvant radiotherapy of the left thigh from July to August 2015 (45 Gy in 25 fractions of 1.8 Gy in 3D conformal radiotherapy), followed by a large resection including the left vastus intermedius, anterior rectus, and part of the vastus lateralis muscles 7 weeks later (October 2015). Surgery was classified as R0.

The extension work-up revealed multiple bilateral pulmonary micronodules that were managed via active surveillance. In January 2016, the progression of these micronodules led to the introduction of the first-line systemic treatment with sunitinib at a dosage of 37.5 mg per day, 3 out of 4 weeks. Co-medications included on-demand inhaled fluticasone/salmeterol and salbutamol. The initial tolerance of the treatment was good, marked by grade 1 hand–foot syndrome, grade 1 nail toxicity (streaks), and grade 1 diarrhea.

**Fig. 2:**
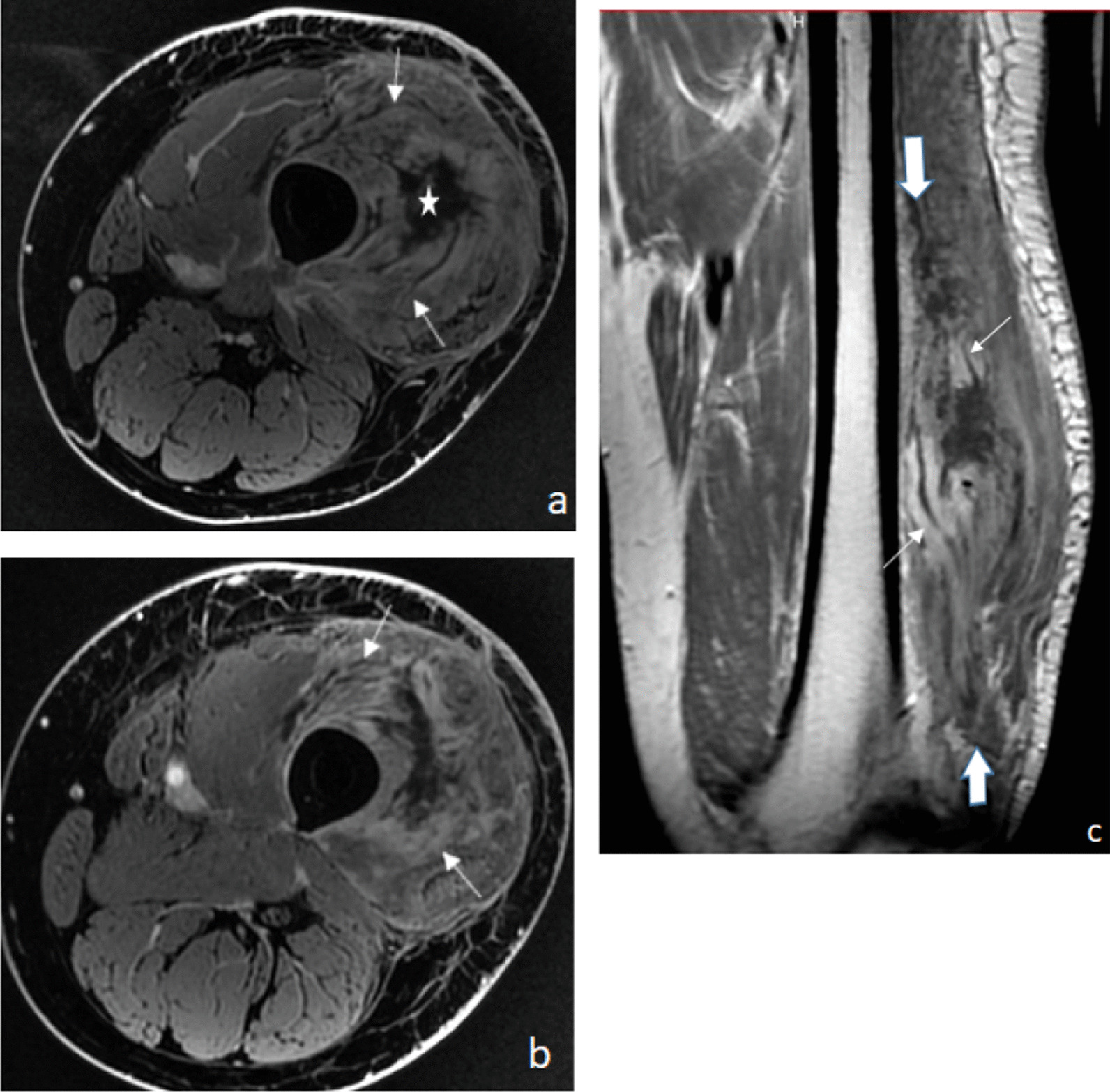
Early-stage axial fat-suppressed T2-weighted MR image (**a**) showing a marked, diffuse, high-signal edema of the soft tissue (arrows) circumscribing a central core (star). Gadolinium-enhanced fat-suppressed T1-weighted MR image (**b**) shows diffuse enhancement of the intermuscular fascia (arrows) without any nodular mass. Coronal T_1_-weighted MR image (**c**) shows extensive infiltration of the soft tissue (big arrows) with convergence of normal muscular fibers (fine arrows) through the central core. *MR* magnetic resonance

In May 2016, 4 months after the first dose of sunitinib, the patient experienced painful swelling in the surgical scar area. Magnetic resonance imaging (MRI) showed diffuse and marked edema of the anterior compartment of the thigh characterized by high signal intensity on T2-weighted images, without nodular lesions, circumscribing a central core, and without any bone signal abnormality. The increased visibility of the intermuscular fascia and the convergence of normal muscular fibers (black-hole effect), in contrast with the displacement seen in tumors, were other characteristics suggestive of myositis (Fig. 2).

**Fig. 3:**
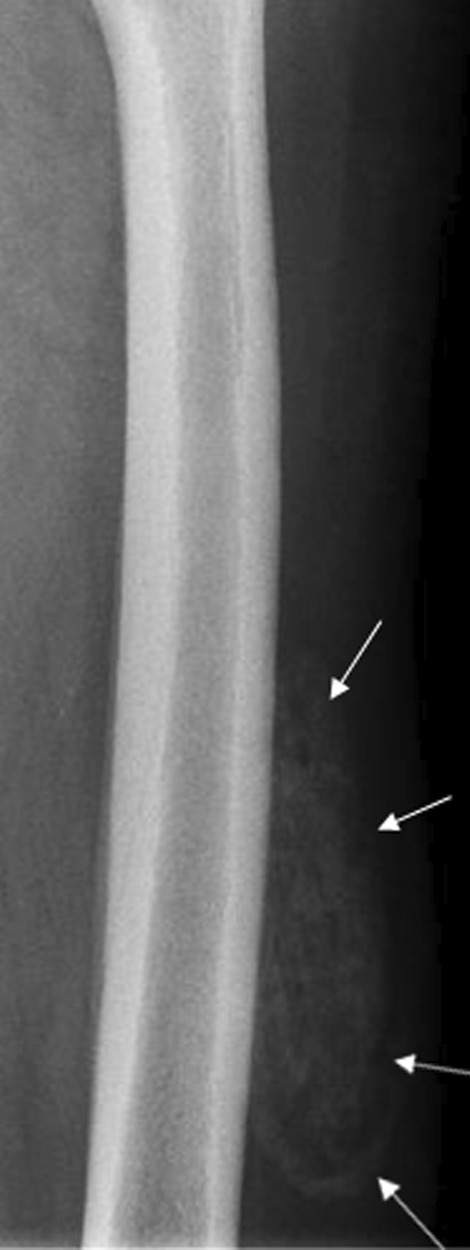
Plain radiograph showing a well-circumscribed mass with an ossified shell (arrows)

Therefore, antiangiogenic treatment was discontinued, and the symptoms rapidly resolved within a few days. Three weeks after the discontinuation of sunitinib, the inflammatory findings completely disappeared. However, a stony-hard zone < 10 cm developed. Six weeks after the diagnosis, plain radiograph showed well-circumscribed ossifications in the soft parts of the external face of the left thigh, extending > 12 cm (Fig. 3). This finding was compatible with a MOC diagnosis.

**Fig. 4:**
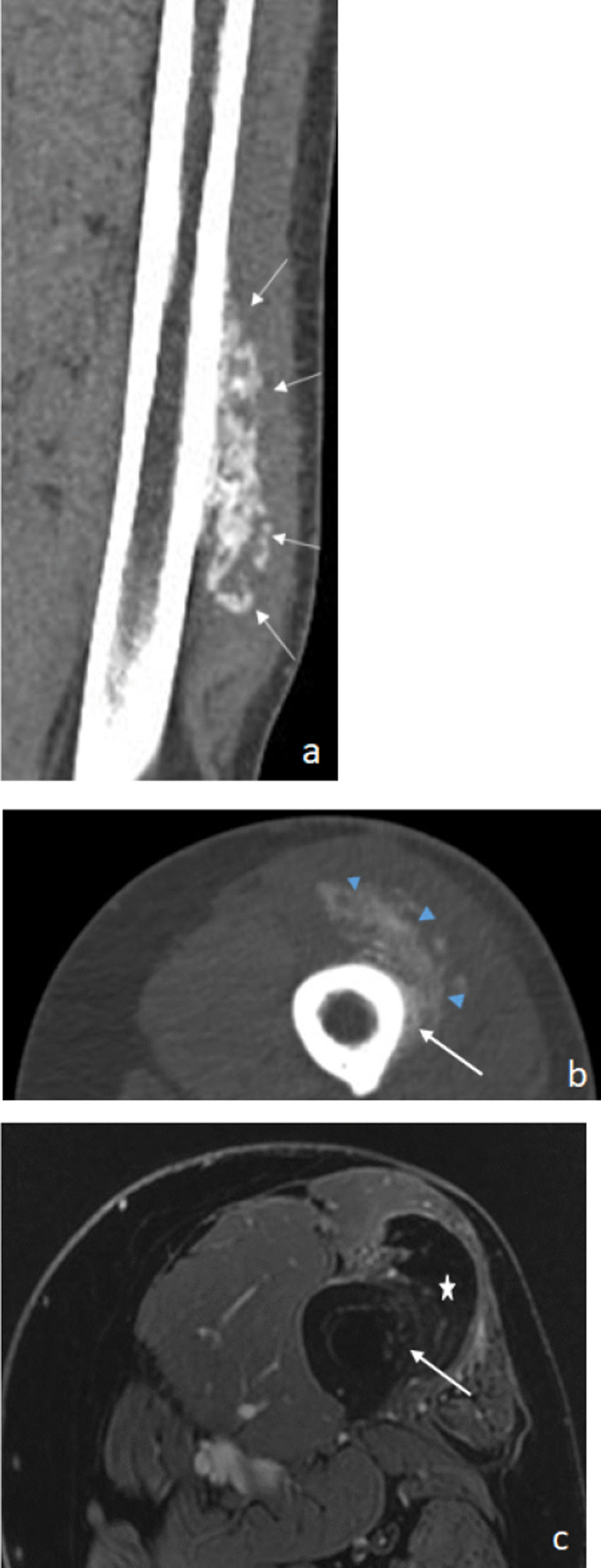
Reconstructed coronal (**a**) and axial (**b**) CT images showing an extensive calcified mass in the anterior muscle compartment (arrowhead) that is continuous with the cortical bone through periosteal ossification (arrow) in some places. Gadolinium-enhanced fat-suppressed T1-weighted MR image (**c**) shows a hypo-intensity T1 lesion without enhancement (star) corresponding to calcification. Note the attachment to the cortical bone (arrow). *CT* computed tomography, *MR* magnetic resonance

In July 2016, 2 months after the diagnosis, computed tomography (CT) showed an extensive calcified mass > 12 cm in height in the anterior muscle compartment, which was continuous with the cortical bone through periosteal ossification in some places. Finally, on MRI, the mass was hypointense without enhancement with gadolinium on T2-weighted images, corresponding to a calcified mass (Fig. 4).

**Fig. 5:**
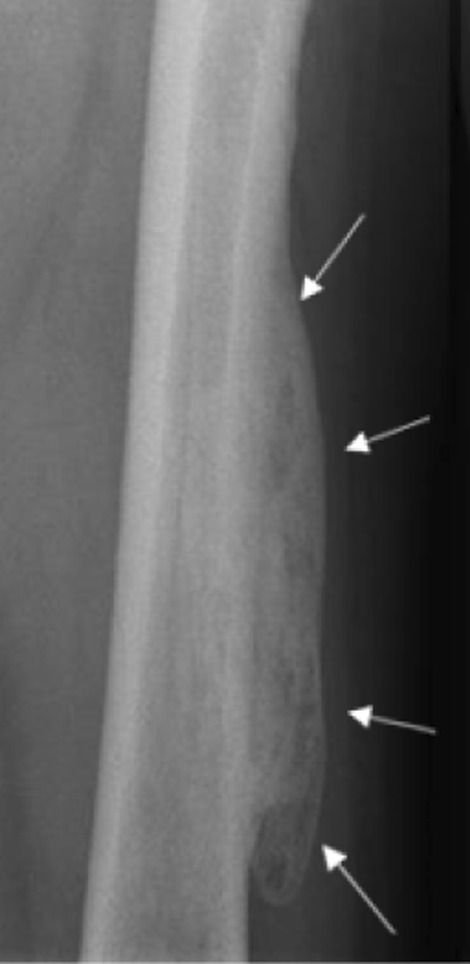
Plain radiograph at the mature stage shows global ossification of the mass (arrows) with complete incorporation into the femoral cortical bone

The continuation of favorable clinical outcomes was confirmed. Five months after the diagnosis of MOC, the patient remained asymptomatic. A plain radiograph showed global ossification of the mass corresponding to a typical MOC fully incorporated into the femoral cortical bone (Fig. 5).

## Discussion and conclusions

### Current management of MOC

The pathophysiology of MOC is poorly understood, but it likely results from the inappropriate differentiation of fibroblasts into osteogenic cells [3]. This may be secondary to the dysregulation of local stem cells in response to tissue injury and subsequent inflammation [4].

The epidemiology of MOC is also poorly understood. It occurs most often in adults aged < 30 years. However, the risk factors are poorly identified. A history of recent trauma has been reported in approximately 50% of cases.

Clinically, MOC presents as a painful swelling, which rapidly increases in size. Additionally, patients may present with associated paresthesia, weakness, lymphedema, and venous thromboembolic disease when the lesion compresses nearby neurovascular structures [3]. Some local inflammatory signs such as redness and heat may be present. Biological inflammatory syndromes may also be associated. This constitutes an “osteogenic mesenchymal storm” [5]. The disease is characterized by its uniqueness and rapid progression.

After approximately 2–6 weeks, the painful and inflammatory symptoms spontaneously disappear, and the mass stabilizes or decreases. This spontaneous evolution distinguishes MOC from other lesions, particularly malignant tumors. This short clinical course helps to avoid unnecessary biopsies or surgery.

Radiologically, there are two phases. The first is the acute phase, which is followed by the mature phase 2–6 weeks later.

In the acute phase, plain radiographs are normal, and ultrasound may show a non-specific hypoechoic area. Magnetic resonance imaging (MRI) shows a T2 hypersignal and clear focal contrast [5]. A characteristic finding that helps differentiate this pathology from malignant soft tissue tumors is the ink “black hole” effect [6] seen on MRI that results from the convergence of normal muscle fibers that appear to be attracted to the lesion. In contrast, malignant soft-tissue tumors displace the surrounding normal muscle fibers.

In the mature phase, the most useful tests are plain radiographs and computed tomography, which show blurred calcifications around a hypodense center. Ultrasonography shows hyperechoic arciform bands located at the periphery of the initial hypoechoic focus. This radiological evolution at 2–6 weeks after presentation is pathognomonic for MOC.

Management is often non-surgical, based on the “RICE” protocol (rest, ice, compression, and elevation) or cryotherapy. In a case report of a football player with post-traumatic MOC in the quadriceps, two doses of pamidronate were associated with improvements in both clinical and imaging features [7]. Surgical excision is reserved for lesions that fail to respond to non-surgical treatment. The optimal timing of surgical excision is unclear but traditionally it is considered to be best performed once the lesion has reached maturity [2].

### Prior case reports

To the best of our knowledge, we cannot find prior descriptions of MOC occurring during sunitinib exposure. We found a letter describing “myositis” in patient treated with sunitinib, but there is no appropriate follow-up that could confirm the diagnosis of MOC [8].

### Accountability of the surgery

Trauma is the most frequently described etiology of MOC. This may be a single trauma or repetitive minor trauma. Here, we considered cancer surgery to be a major trauma. The symptoms of MOC appeared 7 months after surgery.

### Accountability of radiotherapy

To the best of our knowledge, no association between radiotherapy and MOC occurrence has been reported in literature.

### Sunitinib accountability and physiopathology hypothesis

Sunitinib is a tyrosine-kinase inhibitor with several targets (vascular endothelial growth factor receptor, platelet-derived growth factor receptor, KIT, and FLT3) that are involved in tumor growth, pathological neoangiogenesis, and metastatic cancer progression. In preclinical models, platelet-derived growth factor receptor, but above all c-KIT, two well-known targets of sunitinib, play a key role in bone formation, homeostasis of mesenchymal stem cell, and differentiation into osteogenesis progenitors. We hypothesize that in healing scars, sunitinib exposure modified the mesenchymal stem cell differentiation regulation into osteoblast-like cells [9–14]. Another hypothesis is that sunitinib alters mitochondria functions. The accumulated reactive oxygen species can induce myositis secondary to mitochondria damage and dysfunction. In the continuum, mitochondria dysfunction results in the accumulated myoplasmic calcium that induces myositis ossificans [15].

Therefore, the role of sunitinib in the pathogenesis of MOC cannot be excluded. However, MOC development is not a known adverse event with sunitinib in the summary of product characteristics, literature, or French pharmacovigilance database.

Although the pathophysiology of myositis ossificans remains poorly understood, drugs are rarely mentioned among its etiologies. However, according to the international database of the World Health Organization (Vigilyze), among 50 cases of MOC, biphosphonates were involved in 31. In contrast, tyrosine-kinase inhibitors have not been implicated. A previous study mentions drug abuse as a cause of myositis ossificans, but without further precise details [16].

Considering these factors and the timing of events, sunitinib responsiveness cannot be formally excluded.

Herein, we report the case of a patient who developed MOC 7 months after neoadjuvant radiotherapy and surgery for metastatic ASPS. The natural course of MOC does not allow for a clear assessment of sunitinib liability, particularly in the context of a recent history of extensive surgery and radiotherapy.

## Data Availability

The datasets used and analyzed during the current study are available from the corresponding author on reasonable request.

## Abbreviations

ASPS: Alveolar soft part sarcoma
CT: Computed tomography
MOC: Myositis ossificans circumscripta
MRI: Magnetic resonance imaging

## References

[1] Kransdorf MJ, Meis JM, Jelinek JS. Myositis ossificans: MR appearance with radiologic-pathologic correlation. AJR Am J Roentgenol. 1991;157:1243–8. 10.2214/ajr.157.6.1950874. 1950874

[2] Walczak BE, Johnson CN, Howe BM. Myositis ossificans. J Am Acad Orthop Surg. 2015;23:612–22. 10.5435/JAAOS-D-14-00269. 26320160

[3] Mavrogenis AF, Soucacos PN, Papagelopoulos PJ. Heterotopic ossification revisited. Orthopedics. 2011;34:177. 10.3928/01477447-20110124-08. 21410128

[4] Kan L, Liu Y, McGuire TL, Berger DMP, Awatramani RB, Dymecki SM, *et al*. Dysregulation of local stem/progenitor cells as a common cellular mechanism for heterotopic ossification. Stem Cells. 2009;27:150–6. 10.1634/stemcells.2008-0576. PMC2752983 18832590

[5] Wybier M. Myosite ossifiante circonscrite en tomodensitométrie. Instantanés Med. 1988.

[6] Malghem J. Myosite ossifiante circonscrite. In: Conduite à tenir devant une image osseuse des parties molles d’allure tumorale. opus XXXI du GETROA. Sauramps médical; 2004;p. 327–46.

[7] Mani-Babu S, Wolman R, Keen R. Quadriceps traumatic myositis ossificans in a football player: management with intravenous pamidronate. Clin J Sport Med. 2014;24:e56–8. 10.1097/JSM.0000000000000034. 24184852

[8] Suthar RR, Purandare N, Shah S, Agrawal A, Puranik A, Rangarajan V. Sunitinib-induced myositis detected on 18F-FDG PET/CT. Clin Nucl Med. 2022;47:e311–2. 10.1097/RLU.0000000000004035. 35025809

[9] Nagayoshi K, Ohkawa H, Yorozu K, Higuchi M, Higashi S, Kubota N, *et al*. Increased mobilization of c-kit+ Sca-1+ Lin- (KSL) cells and colony-forming units in spleen (CFU-S) following de novo formation of a stem cell niche depends on dynamic, but not stable, membranous ossification. J Cell Physiol. 2006;208:188–94. 10.1002/jcp.20652. 16575918

[10] Wihlidal P, Karlic H, Pfeilstöcker M, Klaushofer K, Varga F. Imatinib mesylate (IM)-induced growth inhibition is associated with production of spliced osteocalcin-mRNA in cell lines. Leuk Res. 2008;32:437–43. 10.1016/j.leukres.2007.07.020. 17822760

[11] Matsumoto T, Ii M, Nishimura H, Shoji T, Mifune Y, Kawamoto A, *et al*. Lnk-dependent axis of SCF-cKit signal for osteogenesis in bone fracture healing. J Exp Med. 2010;207:2207–23. 10.1084/jem.20100321. PMC2947078 20855498

[12] Suphanantachat S, Iwata T, Ishihara J, Yamato M, Okano T, Izumi Y. A role for c-Kit in the maintenance of undifferentiated human mesenchymal stromal cells. Biomaterials. 2014;35:3618–26. 10.1016/j.biomaterials.2014.01.031. 24462355

[13] Liu Q, Zhou Y, Li Z. PDGF-BB promotes the differentiation and proliferation of MC3T3-E1 cells through the Src/JAK2 signaling pathway. Mol Med Rep. 2018;18:3719–26. 10.3892/mmr.2018.9351. PMC6131220 30106097

[14] He DD, Tang XT, Dong W, Cui G, Peng G, Yin X, *et al*. C-KIT expression distinguishes fetal from postnatal skeletal progenitors. Stem Cell Rep. 2020;14:614–30. 10.1016/j.stemcr.2020.03.001. PMC7160391 32220331

[15] Rodríguez-Hernández MA, de la Cruz-Ojeda P, López-Grueso MJ, Navarro-Villarán E, Requejo-Aguilar R, Castejón-Vega B, *et al*. Integrated molecular signaling involving mitochondrial dysfunction and alteration of cell metabolism induced by tyrosine kinase inhibitors in cancer. Redox Biol. 2020;36: 101510. 10.1016/j.redox.2020.101510. PMC7322178 32593127

[16] Aneiros-Fernandez J, Caba-Molina M, Arias-Santiago S, Ovalle F, Hernandez-Cortes P, Aneiros-Cachaza J. Myositis ossificans circumscripta without history of trauma. J Clin Med Res. 2010;2:142–4. 10.4021/jocmr2010.05.364w. PMC3104644 21629528

